# Trend and disparities for smoking during pregnancy in the extreme south of Brazil between 2007 and 2019

**DOI:** 10.1590/1980-549720240055

**Published:** 2024-12-09

**Authors:** Eduardo Peglow, Luana Patrícia Marmitt, Juraci Almeida Cesar

**Affiliations:** I Universidade Federal do Rio Grande, School of Medicine, Graduate Program in Health Sciences – Rio Grande (RS), Brazil.; II Universidade do Oeste de Santa Catarina, Graduate Program in Bioscience and Health – Joaçaba (SC), Brazil.; III Universidade Federal do Rio Grande, School of Medicine, Graduate Program in Public Health – Rio Grande (RS), Brazil.

**Keywords:** Tobacco use disorder, Pregnancy, Time series studies, Health inequities, Sociodemographic factors, Smoking

## Abstract

**Objective:**

To estimate the prevalence and to evaluate trends and disparities in the occurrence of smoking among pregnant women living in the municipality of Rio Grande (RS), in the extreme south of Brazil, between 2007 and 2019.

**Methods:**

All pregnant women living in this municipality who had a child in one of the local hospitals between January 1st and December 31st in the years 2007, 2010, 2013, 2016, and 2019 were included in the study. The interviews took place within 48 hours after childbirth. A pregnant woman was considered a smoker if she smoked at least one cigarette per day for 30 consecutive days in any of the pregnancy trimesters. The respective Pearson's χ^2^ test was used to estimate the proportions and the trend.

**Results:**

The mean prevalence of smoking in the studied period was 17.7% (95%CI 17.0–18.3), dropping from 23.4 (95%CI 21.7–25.0) in 2007 to 12.4% (95%CI 11.1–13.9) in 2019. This decrease occurred in all categories of the studied variables (p>0.001). The greatest disparities in the decrease were observed between the extreme groups for income (75.0 *versus* 34.4%) and level of education (51.0 *versus* 32.1%) and living or not with a partner (50.7 *versus* 27.7%).

**Conclusion:**

There was a sharp and uneven drop in the prevalence of smoking over these 13 years. Pregnant women at higher risk of complications during pregnancy and childbirth were at a clear disadvantage compared to others. Reducing the prevalence of smoking depends on prioritizing interventions among pregnant women with greater social vulnerability.

## INTRODUCTION

Smoking is bad for people's health at any time of their lives. It is the underlying cause of eight million deaths annually worldwide^1^. During pregnancy, it favors the occurrence of ectopic pregnancy^2^, placenta previa^3^, intrauterine growth restriction^4,5^, prematurity^6^, and low birth weight^6,7^, in addition to increasing perinatal and infant morbidity and mortality, due to the increased risk of conditions such as sudden infant death syndrome (SIDS) and neonatal respiratory distress syndrome, and cardiac complications after birth^2,5^.

Globally, the prevalence of smoking during pregnancy varies according to the used definition^8^ and the region, from 0.8% in Africa to 8.1% in Europe. In Brazil, for the country as a whole, the estimated prevalence between 1985 and 2016 was approximately 15%^8^. Authors of a study conducted in the RIPSA (Interagency Health Information Network) cohort found, in Pelotas, state of Rio Grande do Sul (RS) – Brazil, a prevalence of 35.7% in 1982 and 16.5% in 2015; in Ribeirão Preto, state of São Paulo (SP), 28.8% in 1978/1979 and 11.8% in 2010; and in São Luís, state of Maranhão (MA), 6% in 1997/1998 and 4.1% in 2010^9^. Since then, no other representative, time-series study conducted among pregnant women in Brazil has been published. Furthermore, in the aforementioned study that deals with the baseline of the RIPSA cohorts^9^, only Pelotas has two measures for the prevalence of smoking during pregnancy in the 2000s, which is not the most appropriate for assessing trends. Therefore, there is no recent population-based study in Brazil that assesses the tendency towards smoking during pregnancy.

All perinatal studies in the municipality of Rio Grande (RS) were conducted in this century, between 2007 and 2019, with a periodicity of three years, and included all parturients in the municipality, used the same methodology, and had a high respondent rate (98%). Studying the occurrence of smoking over these 13 years can help combating a practice with enormous potential for prevention, especially in prenatal consultations.

We aimed to estimate the prevalence and to evaluate the trend and disparities in the occurrence of smoking during pregnancy in the municipality of Rio Grande (RS), Brazil, between 2007 and 2019.

## METHODS

The municipality of Rio Grande is located in the extreme south of the state of Rio Grande do Sul, 317 km from the state capital, Porto Alegre. Between 2007 and 2019, the period in which these data were collected, its population increased from 195 thousand to 212 thousand inhabitants, the Gross Domestic Product (GDP) increased from BRL 22,300 to BRL 51,900, while the infant mortality rate decreased from 14.8/one thousand to 8.8/one thousand^10^. The health system consists of two hospitals with maternity wards: Hospital Universitário Dr. Miguel Riet Correa Jr. [Dr. Miguel Riet Correa Jr. University Hospital], of Universidade Federal de Rio Grande (HU-FURG), exclusive to the Brazilian Unified Health System (*Sistema Único de Saúde* – SUS), and the Santa Casa de Misericórdia do Rio Grande (Holy House of Mercy of Rio Grande – SCMRG), which serves patients from the SUS, health insurance plans, and private patients. The municipality also has 36 Health Centers (*Unidades Básicas de Saúde* – UBS), with 30 of them offering the Family Health Strategy (*Estratégia Saúde da Família* – ESF), four medical specialty outpatient clinics, and two Emergency Care Units (*Unidades de Pronto Atendimento* – UPA).

The data presented in this article are part of the perinatal studies of Rio Grande, which are regular census surveys conducted every three years since 2007. The target population of these studies represents all parturients in the municipality who gave birth in one of the only two local hospitals between January 1st and December 31st of the years 2007, 2010, 2013, 2016, and 2019. To be included in the study, the mother had to have lived in the municipality for at least six months and her child had to have reached 500 g of birth weight and/or 20 weeks of gestational age.

Data were collected using a single, standardized questionnaire, divided into blocks, with questions ranging from the pre-gestational period to the immediate postpartum period. These are blocks of questions investigating characteristics of the newborn, care received during pregnancy and childbirth, complications during the gestational period, demographic and socioeconomic characteristics of the family as well as lifestyle habits and maternal reproductive history. Whenever possible, variables were collected continuously and subsequently categorized according to analytical needs.

The outcome of this study was smoking during pregnancy. A pregnant woman was considered a smoker if she smoked at least one cigarette per day for 30 consecutive days in any of the pregnancy trimesters.

The variables studied with the aim of assessing disparities were sociodemographic, namely: age, skin color, living with a partner, maternal level of education, and family income. Although most variables are self-explanatory, some of them require additional explanations: family income referred to the sum of the amounts received by all residents of the household in the month immediately prior to the interview. These amounts were obtained in the Brazilian currency, reais (BRL), and, during the analysis, they were converted into minimum wages (MW). Skin color was classified by the very interviewer through observation. Women whose skin color was not Black nor white was classified as brown. Level of education referred to the number of years of studies successfully completed, while adequate prenatal care included starting consultations in the first pregnancy trimester, having six or more consultations, and undergoing at least two tests for HIV, syphilis, and qualitative urine test. This information was obtained from the mother's report and the Pregnant Woman's Card (a document drawn up by the Brazilian Ministry of Health that gathers all information on the pregnancy, childbirth, and the baby).

All puerperae were approached only once, while still in the maternity ward, within 48 hours after giving birth. The visits were carried out daily by interviewers who were duly identified and previously trained to carry out the interviews. Two interviewers worked from Monday to Friday, each in a maternity hospital, and a third one worked on weekends and holidays, rotating between maternity hospitals.

In the 2007, 2010, and 2013 surveys, the interviews were conducted using printed questionnaires and, at the end of each working day, the interviewer reviewed and coded the questionnaires she had applied, delivering them to the study headquarters for later review, double typing, comparison, and correction in the Epidata 3.1 software^11^. In 2016 and 2019, data entry was carried out via tablet, using the REDCap (Research Electronic Data Capture) software^12^, and subsequently downloaded to the FURG server to check for values beyond expectations and other possible inconsistencies.

The consistency analysis, its categorization, and creation of derived variables were performed in the Stata 12 statistical package. The respective Pearson's χ^2^ test was used to estimate the proportions and the trend.

Approximately 10% of the interviews were repeated within two weeks after childbirth. The objective of this stage was to confirm that the interview had been carried out and to assess the agreement between the obtained responses. A reduced questionnaire was used with questions from all blocks of the original questionnaire. The Kappa index varied between 0.60 and 0.99, with most questions being above 0.70, which denotes satisfactory agreement^13^.

The research projects were approved by the FURG Health Research Ethics Committee (CEPAS/FURG) in each of the surveys, under the numbers: 23116.5369/6.58-12/2007, 23116.6258/9.64-117/2009, 23116.2623/67-007-2012, 030/2015, and 278/2018. All participants signed the Informed Consent Form and received a copy of it. Additionally, minor participants signed an Assent Form, and their guardians signed an Informed Consent Form.

## RESULTS

A total of 12,663 puerperae residing in the municipality, who gave birth during the surveyed period, were identified by the Live Birth Information System (*Sistema de Informações sobre Nascidos Vivos* – SINASC). Of these, 12,415 (98%) were successfully interviewed in the five surveys. Losses ranged from 1.3% in 2007 to 2.8% in 2010 and 2013; 0.7%, in 2016; and 2.2%, in 2019. In total, of the five surveys, losses represented 1.9% of the target population.

In Table 1 we show the main demographic, socioeconomic, and reproductive characteristics of these women. Over 13 years, there was a reduction of seven percentage points (p.p.) in the occurrence of births among adolescents, while there was an increase of 9 p.p. among women over 30 years old. The number of white parturients (7 p.p.) who lived with a partner (3 p.p.) also increased. Regarding level of education, we observed a decrease in the lowest stratum (17 p.p.) and an increase among those with 12 or more years of formal education (12 p.p.). The mean family income increased, while the proportion of families with less than one monthly MW (3 p.p.) reduced by 3 p.p., in addition to the reduction of 4 p.p. among families with four or more MW. The participation of mothers in the labor market increased by 5 p.p., and the number of primiparous women (2 p.p.) and multiparous women with three or more children (1 p.p.) decreased. We verified that prenatal care substantially improved during the period, increasing the prenatal care considered adequate by 46 p.p.

**Table 1:** Distribution of puerperae included in perinatal studies according to some characteristics. Rio Grande (RS), 2007–2019.

Characteristics			Survey year % (n)					Total	p-value
			2007	2010	2013	2016	2019	2007–2019	
Maternal age (years)									
	11 to 19		20.4 (515)	18.7 (441)	17.4 (456)	16.9 (448)	13.2 (299)	17.4 (2,159)	<0.001
	20 to 29		52.6 (1,328)	52.6 (1,239)	50.6 (1,324)	49.9 (1,322)	50.5 (1,147)	51.2 (6,360)	
	30 to 47		27.0 (680)	28.7 (675)	32.0 (839)	33.2 (878)	36.3 (824)	31.4 (3,896)	
	Mean (standard deviation)		25.6 (6.6)	25.9 (6.4)	26.2 (6.5)	26.5 (6.6)	26.1 (6.7)	26.3 (6.6)	
Skin color									
	White		69.8 (1,760)	69.6 (1,639)	66.0 (1,728)	67.2 (1,780)	76.4 (1,735)	69.6 (8,642)	<0.001
	Brown		18.3 (462)	20.6 (486)	22.4 (586)	22.6 (598)	15.2 (345)	20.0 (2,477)	
	Black		11.9 (301)	9.8 (230)	11.7 (305)	10.2 (270)	8.4 (190)	10.4 (1,296)	
Lived with a partner									
	Yes		82.6 (2,085)	83.2 (1,960)	85.7 (2,245)	83.7 (2,216)	85.2 (1,933)	84.1 (10,439)	<0.013
	No		17.4 (438)	16.8 (395)	14.3 (374)	16.3 (432)	14.8 (337)	15.9 (1,976)	
Level of education (years)									
	0 to 8		48.8 (1,231)	45.2 (1,065)	39.9 (1,044)	36.7 (972)	31.3 (709)	40.4 (5,021)	<0.001
	9 to 11		41.8 (1,055)	44.5 (1,048)	44.7 (1,171)	39.7 (1,051)	47.2 (1,071)	43.5 (5,396)	
	≥12		9.4 (237)	10.3 (242)	15.4 (404)	23.6 (625)	21.6 (490)	16.1 (1,998)	
Monthly family income in minimum wages									
	<1		12.3 (302)	9.8 (209)	3.5 (89)	5.8 (143)	9.6 (213)	8.1 (956)	<0.001
	1 to 1.9		33.5 (821)	37.2 (794)	29.2 (748)	31.6 (786)	34.6 (765)	33.0 (3,914)	
	2 to 3.9		34.6 (849)	34.2 (729)	40.6 (1,041)	40.5 (1,006)	40.3 (891)	38.1 (4,516)	
	≥4		19.6 (482)	18.9 (403)	26.8 (686)	22.1 (550)	15.5 (342)	20.8 (2,463)	
	Median (BRL)		800.00	1,100.00	1,800.00	2,000.00	2,000.00	1,500.00	
Household's residents									
	2		29.4 (741)	34.2 (805)	34.6 (907)	38.4 (1,017)	26.3 (598)	32.8 (4,068)	<0.001
	3		27.1 (683)	28.5 (672)	30.1 (789)	30.7 (814)	35.2 (799)	30.3 (3,757)	
	≥4		43.6 (1,099)	37.3 (878)	35.2 (923)	30.9 (817)	38.5 (873)	37.0 (4,590)	
	Mean (standard deviation)		3.7 (1.8)	3.5 (1.8)	3.4 (1.7)	3.3 (1.6)	3.5 (1.5)	3.5 (1.7)	
Had a paid work during pregnancy									
	Yes		37.4 (943)	42.9 (1,009)	43.7 (1,145)	45.7 (1,209)	42.5 (965)	42.5 (5,271)	<0.001
	No		62.6 (1,580)	57.2 (1,346)	56.3 (1,474)	54.3 (1,439)	57.5 (1,305)	57.5 (7,144)	
Parity									
	1		39.5 (997)	43.4 (1,023)	47.2 (1,237)	43.3 (1,146)	37.8 (859)	42.4 (5,262)	<0.001
	2		34.5 (871)	36.4 (857)	41.6 (1,089)	35.8 (947)	37.1 (843)	37.1 (4,607)	
	≥3		26.0 (655)	20.2 (475)	11.2 (293)	21.0 (555)	25.0 (568)	20.5 (2,546)	
Received prenatal care deemed adequate									
	Yes		18.2 (459)	40.1 (945)	51.2 (1,341)	49.9 (1,322)	64.0 (1,453)	44.5 (5,520)	<0.001
	No		81.8 (2,064)	59.9 (1,410)	48.8 (1,278)	50.1 (1,326)	36.0 (817)	55.5 (6,895)	
Total		%	20.3	19.0	21.1	21.3	18.3	100.0	
		n	2,523	2,355	2,619	2,648	2,270	12,415	

**Figure 1:**
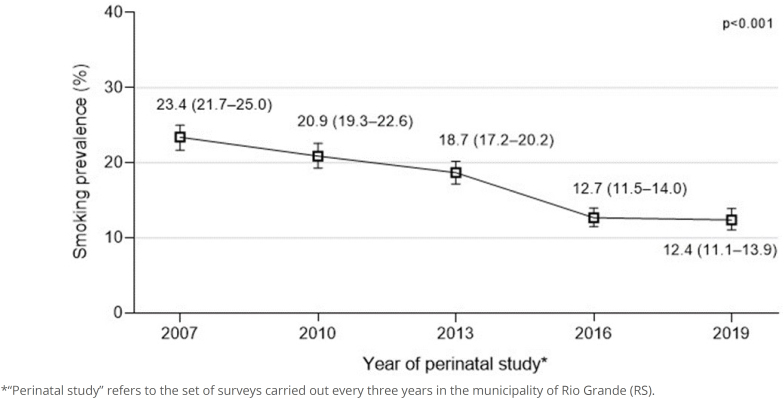
Prevalence of smoking during pregnancy according to the year in which the perinatal survey was conducted. Rio Grande (RS), 2007–2019.

In Figure 1 we show that the average prevalence of smoking in the period was 17.7% (95% confidence interval – 95%CI 17.0–18.3), decreasing from 23.4% (95%CI 21.7–25.0) in 2007 to 12.4% (95%CI 11.1–13.9) in 2019 (a 47% drop). Although the downward trend is significant for the period, it is worth noting that between 2016 and 2019 the reduction in the prevalence of smoking was not significant.

*"Perinatal study" refers to the set of surveys carried out every three years in the municipality of Rio Grande (RS).

In Table 2 we demonstrate smoking trends between 2007–2019 according to the sociodemographic characteristics of puerperae. We observed that the downward trends were significant in all categories of variables throughout the studied period. However, this drop was quite uneven between categories of the same variable. We observed the greatest disparities in the variables "monthly family income" (decrease of 75% between the highest quartile and 34.4% between the lowest quartile), "living with a partner" (decrease of 50.7% among those with a partner and 27.7% among those without a partner), and "level of education" (decrease of 51% among those aged 12 or over and 32.1% among those aged up to 8 years).

**Table 2:** Prevalence of smoking during pregnancy according to sociodemographic characteristics in the municipality of Rio Grande (RS), 2007–2019.

Characteristics			Survey year % (n)					Decrease (%)	Trend p-value
			2007	2010	2013	2016	2019		
Maternal age (years)									
	11 to 19		21.4 (110)	18.4 (81)	14.5 (66)	10.0 (45)	9.0 (27)	57.9	<0.001
	20 to 29		23.9 (317)	22.9 (284)	20.5 (272)	13.2 (175)	13.1 (150)	45.2	
	30 to 47		23.8 (162)	19.0 (128)	18.1 (152)	13.3 (117)	12.7 (105)	46.6	
Skin color									
	White		20.9 (368)	17.0 (279)	15.5 (267)	10.1 (179)	11.0 (190)	47.4	<0.001
	Brown/Black		29.0 (221)	29.9 (214)	25.0 (223)	18.2 (158)	17.2 (92)	40.7	
Lived with a partner									
	Yes		22.1 (460)	18.8 (369)	17.0 (382)	11.6 (257)	10.9 (210)	50.7	<0.001
	No		29.5 (129)	31.4 (124)	28.9 (108)	18.5 (80)	21.4 (74)	27.7	
Level of education (years)									
	0 to 8		34.3 (422)	32.8 (349)	30.8 (322)	22.5 (219)	23.3 (165)	32.1	<0.001
	9 to 11		14.7 (155)	12.4 (130)	12.7 (149)	8.5 (89)	9.8 (105)	33.3	
	≥12		5.1 (12)	5.8 (14)	4.7 (19)	4.6 (29)	2.5 (12)	51.0	
Monthly family income (quartile)									
	1st (lowest)		35.2 (216)	32.3 (177)	28.9 (215)	19.3 (129)	23.1 (131)	34.4	<0.001
	2nd		24.9 (163)	24.2 (131)	19.2 (104)	16.3 (107)	15.1 (87)	39.4	
	3rd		20.4 (117)	17.4 (91)	16.4 (106)	7.9 (58)	7.1 (38)	65.2	
	4th (highest)		12.0 (73)	7.8 (41)	8.0 (501)	3.5 (15)	3.0 (16)	75.0	
Smoking prevalence		%	23.4	20.9	18.7	12.7	12.4	47.0	<0.001
		n	589	493	490	337	282	2,191	

## DISCUSSION

In this study we showed a very sharp drop and great disparity between categories of sociodemographic variables for the occurrence of smoking during pregnancy in the surveyed period.

The prevalence of smoking during the period was reduced by almost half, falling from 23.4% in 2007 to 12.4% in 2019. This pattern is similar to that observed in other studies in Brazil whose authors used the same methodology to define the outcome, such as in Pelotas (RS), where the prevalence fell from 27.7% in 2004 to 16.5% in 2015 (a decrease of 40.4%)^9^. It should be noted that, between 2016 and 2019, this reduction was more slight and not significant, that is, although lower than at the beginning of the studied period, in 2007, it apparently stagnated in the last two surveys.

During the 13 years analyzed, important national tobacco control actions were implemented such as the National Tobacco Control Program (*Programa Nacional de Controle do Tabagismo* – PNCT). The program includes educational, communication, and healthcare actions, promoting cessation and prevention of smoking initiation^14^. In 2007, the year of the first perinatal survey, the Saber Saúde Program (*Programa Saber Saúde*) was established by the National Cancer Institute (*Instituto Nacional de Câncer* – INCA), which contributes to issues related to health promotion and prevention, especially smoking, with the aim of forming critical citizens, capable of deciding on the adoption of healthy behaviors, sharing knowledge with the local community^15^. However, more recently, there has been a weakening of the main action to prevent initiation and encourage cessation, which would be the pricing and tax policy on tobacco products. Indeed, it is no coincidence that for the most recent period, from 2016 to 2019, the most socioeconomically deprived groups showed an increase in the proportion of pregnant women who smoke in this study.

Moreover, regarding the reduction in smoking rates over the period in general, it is worth considering the reduction in Brazil as a whole. The proportion of pregnant women who smoke has decreased by around 50% since the 1980s^6^; likewise, the prevalence of female smokers in the general population has fallen, from 13.9% in 2008 to 9.6% in 2019 (a drop of 30.9%)^16^. Clearly, the results observed for Rio Grande show the same pattern of decline observed in other locations in Brazil. Thus, disproportionate reductions in the proportion of smokers among pregnant women reinforce the inequity in the distribution of the population of female smokers as a whole.

The decrease in the prevalence of smoking — although it had occurred in all categories of the studied variables — did not occur homogeneously, evidencing marked disparities between the subgroups of the analyzed women. Among pregnant adolescents (<20 years old), the drop was 57.9%, compared to 46.6% among those aged ≥30 years. In Spain, between 1990 and 2016, the opposite was observed, that is, a 14.3% drop in the prevalence of smoking among pregnant women under 30 years of age compared to 34.8% among others^17^. This difference in the decrease rate is due to the composition of age groups, the prevalence of smoking, and the periods of comparison. Overall, older women have been dependent for longer, which makes it even more difficult to quit smoking and helps explaining this observed difference^18^. Nonetheless, more recently, it is worth mentioning that younger women have been increasingly exposed to the tobacco industry's marketing strategies — such as the presence of electronic smoking devices and the harm reduction discourse^19^. These products have a strong appeal to young people, which may discourage pregnant women who smoke from quitting smoking and/or encourage young teenagers to develop this habit^20^.

In Rio Grande, the decrease was 40.7% among pregnant women with brown or Black skin color and 47.4% among those with white skin color. In Pelotas, between 2004 and 2011, this drop was 19 and 29.8%, respectively^21^. Apparently, white pregnant women are more aware of the harm caused by smoking, which makes them give up this practice to a greater extent compared to others. Furthermore, it is worth mentioning social inequality associated with racial discrimination as the basis of this process — which, while excluding these women, it pushes them toward practices and lifestyle habits that are harmful to them^22^.

The drop in the prevalence of smoking among pregnant women with a partner was almost double compared to the others (50.7 *versus* 27.7%). Authors of a study conducted in Canada between 1995 and 2010 showed a decrease of 42.2% among those with a partner and 19% among those without a partner^23^. The presence of a partner has the potential to help with household chores, paying family expenses, educating children, ensuring the safety, stability, and well-being of the family, in addition to being able to offer support to the pregnant woman, reducing her stress and anguish and, thus, making her feel safer about giving up a practice that is harmful to her health and that of her family^23,24^.

It is widely known that the higher the level of education and income, the lower the prevalence of smoking during pregnancy^21,25,26^. In Rio Grande, it was no different. While the decrease among those with up to 8 years of formal education was 32.1% in the period, among those with 12 years or more of formal education, it reached 51%. In Pelotas, between 1982 and 2015, there was a 13% increase in the proportion of pregnant smokers with 0–4 years of formal education, while there was a 78% reduction in relation to ≥12 years of formal education^9^. In this same comparison in Ribeirão Preto, between 1978 and 2010, the prevalence did not change among smokers in the first group, but it fell 73% in the second group. In São Luís, a similar result was observed between 1997 and 2010, not changing among those with up to 4 years of formal education and falling 91% among those with ≥12 years^9^. This can be attributed to the adoption of healthy behaviors by the most privileged groups of the population, who have greater access to information, education, and economic resources^21^.

In relation to family income, in Rio Grande, the drop in the lowest quartile was 34.4% and 75% in the highest quartile. In Pelotas, between 2004 and 2011, the reduction in the poorest quintile was 15.3%, while in the richest quintile, 40.5%^21^. This results from the so-called "inverse equity hypothesis," a situation in which new strategies, programs, and interventions first reach those with a higher socioeconomic level and later, the poorest^27^. In addition, the low price charged on the illegal cigarette market ends up favoring initiation and hindering smoking cessation in the population with lower socioeconomic status and lower levels of education. Measures considered effective include increasing taxes, increasing the price of cigarettes, eliminating illicit trade and manufacturing as well as smuggling and counterfeiting^28^.

It is worth highlighting that, during the study period, there was a significant increase in the coverage of prenatal care considered adequate in the sample. Quality prenatal care has the potential to reduce the prevalence of smoking by offering educational interventions and ongoing support, allowing early identification and monitoring of pregnant women who smoke. Health professionals can provide information about the risks of smoking for the mother and fetus, in addition to providing resources for cessation, such as counseling, behavioral therapies and, when necessary, pharmacological treatment, possibly influencing the sample results^29^.

We should mention some study limitations. Studies based on self-reporting, especially when it comes to negative outcomes, such as smoking, may underestimate their real prevalence, as the interviewee tends to hide and deny this practice. The study outcome considered pregnant women who smoked daily for at least one month of pregnancy, which may not have identified occasional smokers. Furthermore, as women with fetal losses (babies weighing less than 500 g at birth and/or less than 20 weeks of gestational age) were not included in the study, there may be an unintentional selection bias in the sample, due to the exclusion of pregnant women who smoked and lost their children during pregnancy. Therefore, the prevalence of smokers could be even higher. Conversely, there are positive points in this study. This is a census study, with few losses in a medium-sized municipality. It should be noted that almost all studies that addressed this topic with pregnant women did so through self-reporting, which ensures the comparability made here. Finally, due to the possible limitations presented, which may vary according to the sociodemographic variables studied, the effect may be to alter the magnitude of reductions over time in the proportion of surveyed smokers.

Overall, smoking, among different groups, is a strategic issue for Brazil in relation to the United Nations 2030 Agenda and the Sustainable Development Goals (SDGs) undertaken by the country, particularly the goals to be achieved in objective 3 — "Health and Well-Being." This objective includes goals, such as reducing maternal and infant mortality, and reducing premature mortality from noncommunicable diseases, such as those associated with smoking. Collecting data on smoking among pregnant women is important to help achieving these goals, identifying more vulnerable groups and socioeconomic inequalities, directing public health interventions to reduce smoking and, consequently, improve maternal and child health^30^.

We showed a sharp drop in the prevalence of smoking and a huge disparity between categories of all variables studied over these 13 years. Based on these results, at least five anti-smoking measures could be adopted, such as:

1. Carry out campaigns in schools, especially high schools, mentioning the harmful effects of smoking;
2. Develop actions to prevent smoking initiation and encourage cessation of this harmful practice in the municipality's Health Centers (UBS), including health professionals, especially community health agents, to work at home level;
3. Continuously publicize that the SUS provides support and treatment for abandoning this practice free of charge;
4. Disseminate, through different means, especially on social media and in the health and education sectors, the benefits of stopping smoking; and
5. Include, in future research on this topic, the use of electronic cigarettes during pregnancy.
