# Supplementary material for: Trend and disparities for smoking during pregnancy in the extreme south of Brazil between 2007 and 2019
Source: Rev Bras Epidemiol. 2024 Dec 9;27:e240055. doi: 10.1590/1980-549720240055 (PMC11656496; doi:10.1590/1980-549720240055)
Supplement: Translation

# Translation: Tendência e disparidades para tabagismo na gestação no extremo sul do Brasil, 2007 a 2019

**DOI:** 10.1590/1980-549720240055.2

**Authors:** Eduardo Peglow, Luana Patrícia Marmitt, Juraci Almeida Cesar

**Affiliations:** I Universidade Federal do Rio Grande, Faculdade de Medicina, Programa de Pós-Graduação em Ciências da Saúde – Rio Grande (RS), Brasil.; II Universidade do Oeste de Santa Catarina, Programa de Pós-Graduação em Biociências e Saúde – Joaçaba (SC), Brasil.; III Universidade Federal do Rio Grande, Faculdade de Medicina, Programa de Pós-Graduação em Saúde Pública – Rio Grande (RS), Brasil.

**Keywords:** Tabagismo, Gravidez, Estudos de séries temporais, Desigualdades de saúde, Fatores sociodemográficos, Fumar

## Abstract

**Objetivo:**

Estimar prevalência, avaliar tendência e disparidades na ocorrência de tabagismo entre gestantes residentes em Rio Grande (RS), entre 2007 e 2019.

**Métodos:**

Foram incluídas nestes estudos todas as gestantes residentes no município que tiveram filho em algum dos hospitais locais entre 1o de janeiro e 31 de dezembro nos anos de 2007, 2010, 2013, 2016 e 2019. As entrevistas ocorreram em até 48 horas após o parto. Considerou-se como tabagista a gestante que fumou pelo menos um cigarro por dia durante 30 dias em algum dos trimestres da gestação. Utilizou-se o respectivo teste χ2 para estimar as proporções e a tendência.

**Resultados:**

A prevalência média de tabagismo no período estudado foi 17,7% (intervalo de confiança de 95% – IC95% 17,0–18,3), caindo de 23,4% (IC95% 21,7–25,0) em 2007 para 12,4% (IC95% 11,1–13,9) em 2019. Essa queda ocorreu em todas as categorias das variáveis estudadas (p<0,001). As maiores disparidades na queda foram observadas entre os grupos extremos para renda (75,0 *versus* 34,4%) e escolaridade (51,0 *versus* 32,1%) e viver ou não com companheiro (50,7 *versus* 27,7%).

**Conclusão:**

Houve queda acentuada e desigual na prevalência de tabagismo ao longo desses 13 anos. Gestantes com maiores riscos de complicação durante a gravidez e o parto estiveram em nítida desvantagem em relação às demais. A redução da prevalência de tabagismo depende de priorizar intervenções entre aquelas de maior vulnerabilidade social.

## INTRODUÇÃO

O tabagismo faz mal à saúde em qualquer momento da vida. É a causa básica de oito milhões de óbitos anualmente em todo o mundo^1^. Na gestação, favorece a ocorrência de gravidez ectópica^2^, placenta prévia^3^, restrição do crescimento intrauterino^4,5^, prematuridade^6^ e baixo peso ao nascer^6,7^, além de aumentar a morbimortalidade perinatal e infantil, pelo aumento do risco de condições, como a síndrome da morte súbita infantil (SMSI) e a síndrome do desconforto respiratório neonatal, e de complicações cardíacas após o nascimento^2,5^.

Em nível mundial, a prevalência de tabagismo na gestação varia de acordo com a definição utilizada^8^ e a região, de 0,8% na África a 8,1% na Europa. No Brasil, para o país como um todo, a prevalência estimada entre 1985 e 2016 foi de aproximadamente 15%^8^. Estudo conduzido na coorte RIPSA encontrou, em Pelotas, Rio Grande do Sul (RS), prevalência de 35,7% em 1982 e 16,5% em 2015; em Ribeirão Preto, São Paulo (SP), 28,8% em 1978/1979 e 11,8% em 2010; e em São Luís, Maranhão (MA), 6% em 1997/1998 e 4,1% em 2010^9^. Desde então, nenhum outro estudo representativo, de série temporal conduzido entre gestantes no Brasil, foi publicado. Além disso, no estudo citado que trata do *baseline* das coortes RIPSA^9^, somente Pelotas dispõe de duas medidas para a prevalência de tabagismo na gestação nos anos 2000, o que não é o mais apropriado para avaliar tendência. Logo, não há, no Brasil, um estudo de base populacional, mais recente, que avalia a tendência para tabagismo no período gestacional.

Todos os estudos perinatais de Rio Grande foram conduzidos neste século, entre 2007 e 2019, com periodicidade de 3 anos, incluíram todas as parturientes do município, utilizaram a mesma metodologia e tiveram alta taxa de respondentes (98%). Estudar a ocorrência de tabagismo ao longo desses 13 anos pode auxiliar no combate a uma prática com enorme potencial de prevenção, sobretudo nas consultas de pré-natal.

O presente estudo teve por objetivos estimar a prevalência, avaliar a tendência e disparidades na ocorrência de tabagismo durante a gestação no município de Rio Grande (RS), entre 2007 e 2019.

## MÉTODOS

O município de Rio Grande localiza-se no extremo sul do Rio Grande do Sul, a 317 km da capital do estado, Porto Alegre. Entre 2007 e 2019, período em que foram coletados esses dados, sua população passou de 195 mil para 212 mil habitantes, o Produto Interno Bruto (PIB) aumentou de R$ 22,3 mil para R$ 51,9 mil, enquanto a taxa de mortalidade infantil caiu de 14,8/1.000 para 8,8/1.000^10^. O sistema de saúde é constituído de dois hospitais com maternidade: Hospital Universitário Dr. Miguel Riet Correa Jr., da Universidade Federal de Rio Grande (HU-FURG), exclusivo do Sistema Único de Saúde (SUS), e a Santa Casa de Misericórdia do Rio Grande (SCMRG), que atende pacientes do SUS, de convênios e particular. O município tem ainda 36 Unidades Básicas de Saúde (UBS), com 30 delas oferecendo a Estratégia Saúde da Família (ESF), quatro ambulatórios de especialidades médicas e duas Unidades de Pronto Atendimento (UPA).

Os dados aqui apresentados fazem parte dos estudos perinatais de Rio Grande, que são inquéritos censitários regulares, conduzidos a cada 3 anos desde 2007. A população alvo desses estudos representa todas as parturientes do município que tiveram parto em algum dos dois únicos hospitais locais entre 1^o^ de janeiro e 31 de dezembro dos anos de 2007, 2010, 2013, 2016 e 2019. Para ser incluída no estudo, a mãe deveria residir há pelo menos 6 meses no município e o seu filho ter alcançado 500 g de peso ao nascer e/ou 20 semanas de idade gestacional.

A coleta de dados foi realizada por meio de questionário único, padronizado, dividido em blocos, com perguntas que incluíam desde o período pré-gestacional até o pós-parto imediato. São blocos de perguntas investigando características do recém-nascido, assistência recebida durante a gestação e o parto, intercorrências no período gestacional, características demográficas e socioeconômicas da família, além de hábitos de vida e história reprodutiva materna. Sempre que possível, as variáveis eram coletadas na forma contínua e, posteriormente, categorizadas conforme a necessidade analítica.

O desfecho deste estudo foi constituído do tabagismo durante a gestação. Considerou-se como tabagista a gestante que fumou pelo menos um cigarro por dia durante 30 dias em algum dos trimestres da gestação.

As variáveis estudadas com o objetivo de avaliar disparidades foram as sociodemográficas: idade, cor da pele, viver com companheiro, escolaridade materna e renda familiar. Apesar de a maioria das variáveis ser autoexplicativa, algumas delas necessitam de explicações adicionais: renda familiar referiu-se à soma dos valores recebidos por todos os moradores do domicílio no mês imediatamente anterior à entrevista. Esses valores foram obtidos em reais e, depois, por ocasião da análise, convertidos em salários mínimos (SM). A cor da pele foi classificada pela própria entrevistadora mediante observação. De acordo com o manual de instruções, aquela que não era de cor da pele preta ou branca foi classificada como parda. Escolaridade referiu-se ao número de anos de estudos completados com aprovação, enquanto a realização de pré-natal adequado compreendia iniciar as consultas no primeiro trimestre de gestação, realizar seis ou mais consultas e ser submetida a pelo menos dois testes para HIV, sífilis e qualitativo de urina. Essas informações foram obtidas a partir do relato da mãe e da Carteira da Gestante.

Todas as puérperas foram abordadas uma única vez, ainda na maternidade, em até 48 horas após o parto. As visitas eram realizadas diariamente pelas entrevistadoras devidamente identificadas e previamente treinadas para realização das entrevistas. Duas entrevistadoras atuaram de segunda a sexta-feira, cada uma em uma maternidade, e uma terceira atuava nos finais de semana e feriados, com rodízio entre as maternidades.

Nos inquéritos de 2007, 2010 e 2013, as entrevistas foram realizadas em questionários impressos e, ao final de cada dia de trabalho, a entrevistadora revisava e codificava os questionários por ela aplicados, entregando-os na sede do estudo, para posterior revisão, dupla digitação, comparação e correção no *software* Epidata 3.1^11^. Em 2016 e 2019, a entrada de dados foi feita via *tablet*, utilizando-se o *software* REDCap (Research Electronic Data Capture)^12^, e, posteriormente, descarregada no servidor da FURG para verificação de valores além do esperado e outras eventuais inconsistências.

A análise de consistência, sua categorização e criação de variáveis derivadas foram feitas no pacote estatístico Stata 12. Utilizou-se o respectivo teste χ^2^ para estimar as proporções e a tendência.

Cerca de 10% das entrevistas foram refeitas em até duas semanas após o parto. O objetivo dessa etapa era confirmar a realização da entrevista e avaliar a concordância entre as respostas obtidas. Utilizou-se questionário reduzido com perguntas de todos os blocos do questionário original. O índice Kappa variou entre 0,60 e 0,99, com a maioria das questões ficando acima de 0,70, o que denota concordância satisfatória^13^.

Os projetos de pesquisa foram aprovados pelo Comitê de Ética em Pesquisa na Área da Saúde da FURG (CEPAS/FURG) em cada um dos inquéritos, sob os números: 23116.5369/6.58-12/2007, 23116.6258/9.64-117/2009, 23116.2623/67-007-2012, 030/2015 e 278/2018. Todas as participantes assinaram o Termo de Consentimento Livre e Esclarecido (TCLE) e receberam uma cópia dele. Adicionalmente, participantes menores de idade assinaram um termo de assentimento, e os seus responsáveis, um TCLE.

## RESULTADOS

Foram identificadas pelo Sistema de Informações sobre Nascidos Vivos (SINASC) 12.663 puérperas residentes no município com parto no período estudado. Destas, 12.415 (98%) foram entrevistadas com sucesso nos cinco inquéritos. As perdas variaram em 1,3%, no ano de 2007, a 2,8%, nos anos de 2010 e 2013, 0,7% em 2016 e 2,2% em 2019. No total, dos cinco inquéritos, as perdas representaram 1,9% da população alvo.

A Tabela 1 exibe as principais características demográficas, socioeconômicas e reprodutivas dessas mulheres. Ao longo de 13 anos, houve redução de 7 pontos percentuais (p.p.) na ocorrência de partos entre adolescentes, ao mesmo tempo em que houve aumento de 9 p.p. entre mulheres com mais de 30 anos. Também aumentou o número de parturientes brancas (7 p.p.), que viviam com companheiro (3 p.p.). Quanto à escolaridade, observou-se redução no menor estrato (17 p.p.) e aumento entre aquelas com 12 anos ou mais de estudo (12 p.p.). A renda familiar média aumentou, enquanto reduziu em 3 p.p. a proporção de famílias com menos de um SM mensal (3 p.p.), além da redução de 4 p.p. entre famílias com 4 ou mais SM. A participação das mães no mercado de trabalho aumentou em 5 p.p., e reduziu-se o número de primíparas (2 p.p.) e multíparas com três filhos ou mais (1 p.p.). Verifica-se que a assistência pré-natal melhorou substancialmente no período, aumentando em 46 p.p. o pré-natal considerado adequado.

**Tabela 1:** Distribuição das puérperas incluídas nos estudos perinatais de acordo com algumas características. Rio Grande (RS), 2007–2019.

Características			Ano do inquérito % (n)					Total	p-valor
			2007	2010	2013	2016	2019	2007–2019	
Idade materna (anos)									
	11 a 19		20,4 (515)	18,7 (441)	17,4 (456)	16,9 (448)	13,2 (299)	17,4 (2.159)	<0,001
	20 a 29		52,6 (1.328)	52,6 (1.239)	50,6 (1.324)	49,9 (1.322)	50,5 (1.147)	51,2 (6.360)	
	30 a 47		27,0 (680)	28,7 (675)	32,0 (839)	33,2 (878)	36,3 (824)	31,4 (3.896)	
	Média (desvio padrão)		25,6 (6,6)	25,9 (6,4)	26,2 (6,5)	26,5 (6,6)	26,1 (6,7)	26,3 (6,6)	
Cor da pele									
	Branca		69,8 (1.760)	69,6 (1.639)	66,0 (1.728)	67,2 (1.780)	76,4 (1.735)	69,6 (8.642)	<0,001
	Parda		18,3 (462)	20,6 (486)	22,4 (586)	22,6 (598)	15,2 (345)	20,0 (2.477)	
	Preta		11,9 (301)	9,8 (230)	11,7 (305)	10,2 (270)	8,4 (190)	10,4 (1.296)	
Vivia com companheiro									
	Sim		82,6 (2.085)	83,2 (1.960)	85,7 (2.245)	83,7 (2.216)	85,2 (1.933)	84,1 (10.439)	<0,013
	Não		17,4 (438)	16,8 (395)	14,3 (374)	16,3 (432)	14,8 (337)	15,9 (1.976)	
Escolaridade (anos)									
	0 a 8		48,8 (1.231)	45,2 (1.065)	39,9 (1.044)	36,7 (972)	31,3 (709)	40,4 (5.021)	<0,001
	9 a 11		41,8 (1.055)	44,5 (1.048)	44,7 (1.171)	39,7 (1.051)	47,2 (1.071)	43,5 (5.396)	
	≥12		9,4 (237)	10,3 (242)	15,4 (404)	23,6 (625)	21,6 (490)	16,1 (1.998)	
Renda familiar mensal, em salários mínimos									
	<1		12,3 (302)	9,8 (209)	3,5 (89)	5,8 (143)	9,6 (213)	8,1 (956)	<0,001
	1 a 1,9		33,5 (821)	37,2 (794)	29,2 (748)	31,6 (786)	34,6 (765)	33,0 (3.914)	
	2 a 3,9		34,6 (849)	34,2 (729)	40,6 (1.041)	40,5 (1.006)	40,3 (891)	38,1 (4.516)	
	≥4		19,6 (482)	18,9 (403)	26,8 (686)	22,1 (550)	15,5 (342)	20,8 (2.463)	
	Mediana (R$)		800,00	1.100,00	1.800,00	2.000,00	2.000,00	1.500,00	
Moradores no domicílio									
	2		29,4 (741)	34,2 (805)	34,6 (907)	38,4 (1.017)	26,3 (598)	32,8 (4.068)	<0,001
	3		27,1 (683)	28,5 (672)	30,1 (789)	30,7 (814)	35,2 (799)	30,3 (3.757)	
	≥4		43,6 (1.099)	37,3 (878)	35,2 (923)	30,9 (817)	38,5 (873)	37,0 (4.590)	
	Média (desvio padrão)		3,7 (1,8)	3,5 (1,8)	3,4 (1,7)	3,3 (1,6)	3,5 (1,5)	3,5 (1,7)	
Exerceram trabalho remunerado na gestação									
	Sim		37,4 (943)	42,9 (1.009)	43,7 (1.145)	45,7 (1.209)	42,5 (965)	42,5 (5.271)	<0,001
	Não		62,6 (1.580)	57,2 (1.346)	56,3 (1.474)	54,3 (1.439)	57,5 (1.305)	57,5 (7.144)	
Paridade									
	1		39,5 (997)	43,4 (1.023)	47,2 (1.237)	43,3 (1.146)	37,8 (859)	42,4 (5.262)	<0,001
	2		34,5 (871)	36,4 (857)	41,6 (1.089)	35,8 (947)	37,1 (843)	37,1 (4.607)	
	≥3		26,0 (655)	20,2 (475)	11,2 (293)	21,0 (555)	25,0 (568)	20,5 (2.546)	
Realizou pré-natal considerado adequado									
	Sim		18,2 (459)	40,1 (945)	51,2 (1.341)	49,9 (1.322)	64,0 (1.453)	44,5 (5.520)	<0,001
	Não		81,8 (2.064)	59,9 (1.410)	48,8 (1.278)	50,1 (1.326)	36,0 (817)	55,5 (6.895)	
Total		%	20,3	19,0	21,1	21,3	18,3	100,0	
		n	2.523	2.355	2.619	2.648	2.270	12.415	

A Figura 1 mostra que a prevalência média de tabagismo no período foi 17,7% (intervalo de confiança de 95% – IC95% 17,0–18,3), caindo de 23,4% (IC95% 21,7–25,0) em 2007 para 12,4% (IC95% 11,1–13,9) em 2019 (queda de 47%). Embora a tendência de redução seja significativa para o período, destaca-se que entre 2016 e 2019 a redução na prevalência do tabagismo não foi significativa.

**Figura 1:** Prevalência de tabagismo na gestação conforme o ano de realização do inquérito perinatal. Rio Grande (RS), 2007–2019.

*"Estudo perinatal" refere-se ao conjunto de inquéritos realizados a cada três anos no município de Rio Grande (RS).

A Tabela 2 apresenta tendência ao tabagismo entre 2007–2019 conforme características sociodemográficas das puérperas. Observou-se que as tendências de redução foram significativas em todas as categorias das variáveis ao longo do período estudado. Porém essa queda foi bastante desigual entre categorias da mesma variável. As maiores disparidades foram observadas nas variáveis "renda familiar mensal" (redução de 75% entre o maior quartil e 34,4% entre o menor quartil), "viver com companheiro" (queda de 50,7% entre aquelas com companheiro e 27,7% entre as sem companheiro) e "escolaridade" (queda de 51% entre aquelas com 12 anos ou mais e 32,1% entre aquelas com até 8 anos).

**Tabela 2:** Prevalência de tabagismo durante a gestação conforme características sociodemográficas no município de Rio Grande (RS), 2007–2019.

Características			Ano do inquérito % (n)					Redução (%)	p-valor de tendência
			2007	2010	2013	2016	2019		
Idade materna (anos)									
	11 a 19		21,4 (110)	18,4 (81)	14,5 (66)	10,0 (45)	9,0 (27)	57,9	<0,001
	20 a 29		23,9 (317)	22,9 (284)	20,5 (272)	13,2 (175)	13,1 (150)	45,2	
	30 a 47		23,8 (162)	19,0 (128)	18,1 (152)	13,3 (117)	12,7 (105)	46,6	
Cor da pele									
	Branca		20,9 (368)	17,0 (279)	15,5 (267)	10,1 (179)	11,0 (190)	47,4	<0,001
	Parda/Preta		29,0 (221)	29,9 (214)	25,0 (223)	18,2 (158)	17,2 (92)	40,7	
Vivia com companheiro									
	Sim		22,1 (460)	18,8 (369)	17,0 (382)	11,6 (257)	10,9 (210)	50,7	<0,001
	Não		29,5 (129)	31,4 (124)	28,9 (108)	18,5 (80)	21,4 (74)	27,7	
Escolaridade (anos)									
	0 a 8		34,3 (422)	32,8 (349)	30,8 (322)	22,5 (219)	23,3 (165)	32,1	<0,001
	9 a 11		14,7 (155)	12,4 (130)	12,7 (149)	8,5 (89)	9,8 (105)	33,3	
	≥12		5,1 (12)	5,8 (14)	4,7 (19)	4,6 (29)	2,5 (12)	51,0	
Renda familiar mensal (quartis)									
	1º (menor)		35,2 (216)	32,3 (177)	28,9 (215)	19,3 (129)	23,1 (131)	34,4	<0,001
	2º		24,9 (163)	24,2 (131)	19,2 (104)	16,3 (107)	15,1 (87)	39,4	
	3º		20,4 (117)	17,4 (91)	16,4 (106)	7,9 (58)	7,1 (38)	65,2	
	4º (maior)		12,0 (73)	7,8 (41)	8,0 (501)	3,5 (15)	3,0 (16)	75,0	
Prevalência de tabagismo		%	23,4	20,9	18,7	12,7	12,4	47,0	<0,001
		n	589	493	490	337	282	2.191	

## DISCUSSÃO

Este estudo mostrou queda bastante acentuada e grande disparidade entre categorias das variáveis sociodemográficas para ocorrência de tabagismo durante a gestação no período estudado.

A prevalência de tabagismo no período foi reduzida praticamente à metade, caindo de 23,4% em 2007 para 12,4% em 2019. Esse padrão é similar ao observado em outros estudos no Brasil que utilizaram a mesma metodologia para definição do desfecho, como em Pelotas, RS, onde a prevalência caiu de 27,7% em 2004 para 16,5% em 2015 (queda de 40,4%)^9^. Ressalta-se que, entre 2016 e 2019, essa redução foi mais discreta e não significativa, ou seja, embora mais baixa que no início do período estudado, de 2007, aparentemente estagnou nos dois últimos inquéritos.

Durante os 13 anos analisados, foram implementadas ações nacionais importantes de controle do tabaco, como o Programa Nacional de Controle do Tabagismo (PNCT). O programa contempla ações educativas, de comunicação e de atenção à saúde, promovendo a cessação e a prevenção da iniciação do tabagismo^14^. Em 2007, ano do primeiro inquérito perinatal, foi instituído o Programa Saber Saúde, do Instituto Nacional de Câncer (INCA), que contribui com as temáticas referentes à promoção da saúde e à prevenção, especialmente do tabagismo, com o objetivo de formar cidadãos críticos, capazes de decidir sobre a adoção de comportamentos saudáveis, disseminando os saberes para a comunidade local^15^. No entanto, mais recentemente, observa-se um enfraquecimento da principal ação de prevenção da iniciação e estímulo da cessação, que seria a política de preços e impostos sobre produtos derivados do tabaco. De fato, não por acaso, para o período mais recente, de 2016 a 2019, os grupos mais desprovidos do ponto de vista socioeconômico apresentaram um aumento na proporção de mulheres grávidas fumantes neste estudo.

Outro ponto importante a considerar sobre a redução das taxas de tabagismo no período como um todo é a redução no Brasil como um todo. A proporção de gestantes fumantes diminuiu cerca de 50% desde a década de 1980^6^, da mesma forma que caiu a prevalência de mulheres fumantes na população geral, de 13,9% em 2008 para 9,6% em 2019 (queda de 30,9%)^16^. Claramente, os resultados observados para o Rio Grande apresentam o mesmo padrão de queda observado em outras localidades do Brasil. Assim, as reduções desproporcionais na proporção de fumantes entre mulheres grávidas reforçam a iniquidade na distribuição da população de mulheres fumantes como um todo.

A redução na prevalência do tabagismo, embora tenha ocorrido em todas as categorias das variáveis estudadas, não ocorreu de forma homogênea, revelando disparidades marcantes entre os subgrupos de mulheres analisadas. Entre gestantes adolescentes (<20 anos), a queda foi de 57,9%, contra 46,6% entre aquelas com ≥30 anos. Na Espanha, entre 1990 e 2016, observou-se o inverso, ou seja, queda de 14,3% na prevalência de tabagismo entre gestantes com menos de 30 anos contra 34,8% nas demais^17^. Essa diferença na taxa de queda decorre da constituição dos grupos etários, da prevalência de tabagismo e dos períodos de comparação. Mulheres mais velhas, em geral, têm maior tempo de dependência, o que dificulta ainda mais cessar o tabagismo e ajuda explicar essa diferença observada^18^. No entanto, mais recentemente, não se pode ignorar que mulheres mais jovens têm sido cada vez mais expostas às estratégias de marketing da indústria do tabaco, como a presença dos dispositivos eletrônicos para fumar e o discurso de redução de danos^19^. Esses produtos têm forte apelo ao público jovem, o que pode desestimular a mulher gestante fumante a parar de fumar e/ou incentivar novas adolescentes a iniciar esse hábito^20^.

Em Rio Grande, a queda foi de 40,7% entre gestantes da cor da pele parda ou preta e de 47,4% naquelas de cor da pele branca. Em Pelotas, entre 2004 e 2011, essa redução foi de 19 e 29,8%, respectivamente^21^. Ao que parece, gestantes de cor da pele branca estão mais cientes dos prejuízos causados pelo fumo, o que as faz abandonar essa prática em maior proporção em relação às demais. Além disso, não se pode deixar de citar a desigualdade social, associada à discriminação racial, como a base desse processo que, ao mesmo tempo que as exclui, as empurra em direção a práticas e hábitos de vida que lhes são prejudiciais^22^.

A queda na prevalência de tabagismo entre gestantes com companheiro foi quase o dobro em comparação às demais (50,7 *versus* 27,7%). Estudo conduzido no Canadá, entre 1995 e 2010, mostrou queda de 42,2% entre aquelas com companheiro e de 19% entre as sem companheiro^23^. A presença de companheiro tem potencial para ajudar nos afazeres da casa, no pagamento das despesas da família, na educação dos filhos, na segurança, na estabilidade e no bem-estar da família, além de poder oferecer apoio à gestante, diminuindo o seu estresse e a sua angústia e, assim, fazê-la se sentir mais segura para o abandono de uma prática que faz mal à sua saúde e a da sua família^23,24^.

É amplamente sabido que quanto maior a escolaridade e a renda, menor é a prevalência de tabagismo na gestação^21,25,26^. Em Rio Grande, não foi diferente. Enquanto a queda entre aquelas com até 8 anos de escolaridade foi de 32,1% no período, entre aquelas com 12 anos ou mais de escolaridade, alcançou 51%. Em Pelotas, entre 1982 e 2015, houve aumento de 13% na proporção de tabagistas gestantes com escolaridade de 0–4 anos, ao mesmo tempo em que houve redução de 78% em relação a ≥12 anos de escolaridade^9^. Nessa mesma comparação em Ribeirão Preto, entre 1978 e 2010, a prevalência não se modificou entre tabagistas do primeiro grupo, mas caiu 73% no segundo grupo. Em São Luís, resultado semelhante foi observado entre 1997 e 2010, não se alterando entre aquelas com até 4 anos de estudo e caindo 91% entre aquelas com ≥12 anos^9^. Isso pode ser atribuído à adoção de comportamentos saudáveis pelos grupos mais favorecidos da população, que têm maior acesso à informação, à educação e aos recursos econômicos^21^.

Em relação à renda familiar, em Rio Grande, a queda no menor quartil foi de 34,4% e de 75% no maior quartil. Em Pelotas, entre 2004 e 2011, a redução no quintil mais pobre foi de 15,3%, enquanto no quintil mais rico, 40,5%^21^. Isso decorre da chamada "hipótese da equidade inversa", situação em que novas estratégias, programas e intervenções alcançam primeiro aqueles de melhor nível socioeconômico para somente depois chegarem aos mais pobres^27^. Além disso, o baixo preço praticado no mercado ilegal dos cigarros acaba favorecendo a iniciação e atrapalhando a cessação de tabagismo na população de pior nível socioeconômico e menor escolaridade. Medidas consideradas efetivas incluem aumento no valor dos impostos, no preço dos cigarros, na eliminação do comércio e na fabricação ilícita, bem como em seu contrabando e falsificação^28^.

Cabe destacar que, no período do estudo, houve um aumento significativo na cobertura do pré-natal considerado adequado na amostra. A assistência pré-natal de qualidade tem o potencial de reduzir a prevalência de tabagismo ao oferecer intervenções educativas e suporte contínuo, permitindo a identificação precoce e o acompanhamento das gestantes fumantes. Profissionais de saúde podem fornecer informações sobre os riscos do tabagismo para a mãe e o feto, além de disponibilizar recursos para a cessação, como aconselhamento, terapias comportamentais e, quando necessário, tratamento farmacológico, possivelmente influenciando os resultados na amostra^29^.

Algumas limitações deste estudo precisam ser mencionadas. Estudos baseados no autorrelato, principalmente quando se trata de desfechos negativos, como o tabagismo, podem subestimar a sua real prevalência, porque a entrevistada tende a esconder, negar essa prática. O desfecho do estudo considerou gestantes que fumaram diariamente durante pelo menos um mês de gestação, o que pode não ter identificado as fumantes ocasionais. Além disso, como não foram incluídas na pesquisa mulheres com perdas fetais (bebês com menos de 500 g ao nascer e/ou menos de 20 semanas de idade gestacional), pode haver um viés de seleção não intencional na amostra, em razão da exclusão de gestantes fumantes que perderam seus filhos durante a gestação. Dessa forma, a prevalência de fumantes poderia ser ainda maior. Por outro lado, este estudo apresenta pontos positivos. Trata-se de um estudo censitário, com poucas perdas em um município de porte médio. Vale destacar ainda que a quase totalidade dos estudos que abordaram esse tema junto às gestantes o fez por autorrelato, o que assegura a comparabilidade aqui feita. Por fim, em função das possíveis limitações presentes, que podem variar conforme as variáveis sociodemográficas estudadas, o efeito pode ser o de alterar a magnitude das reduções ao longo do tempo na proporção de fumantes estudadas.

No geral, o tabagismo, nos diferentes públicos, é um tema estratégico para o Brasil perante a relação com a Agenda 2030 das Nações Unidas e os Objetivos de Desenvolvimento Sustentável (ODS) assumidos pelo país, particularmente as metas a serem atingidas no objetivo 3 — "Saúde e Bem-Estar". Este objetivo inclui metas como reduzir a mortalidade materna e infantil, e diminuir a mortalidade prematura por doenças não transmissíveis, como aquelas associadas ao tabagismo. Levantar dados sobre o tabagismo em gestantes é importante para contribuir com o alcance dessas metas, identificando grupos mais vulneráveis e desigualdades socioeconômicas, direcionando intervenções de saúde pública para reduzir o tabagismo e, consequentemente, melhorar a saúde materno-infantil^30^.

Este estudo mostrou queda acentuada na prevalência de tabagismo e enorme disparidade entre categorias de todas as variáveis estudadas ao longo desses 13 anos. A partir desses resultados, pelo menos cinco medidas antitabágicas poderiam ser adotadas, por exemplo:

1. Realizar companhas nos colégios, sobretudo de ensino médio, mencionando os malefícios do fumo;
2. Desenvolver ações preventivas de iniciação ao tabagismo e de encorajamento à cessação dessa prática nociva à saúde nas UBS do município, incluindo profissionais da saúde, sobretudo os agentes comunitários de saúde, para atuar em nível domiciliar;
3. Divulgar continuamente que o SUS disponibiliza, de maneira gratuita, apoio e tratamento para o abandono dessa prática;
4. Disseminar, por diferentes meios, sobretudo nas mídias sociais e nos setores de saúde e educação, os benefícios decorrentes da interrupção do tabagismo;
5. Incluir, em futuras pesquisas sobre esse tema, o uso de cigarros eletrônicos no período gestacional.
